# Taking action on the social determinants of health: improving health access for the urban poor in Mongolia

**DOI:** 10.1186/1475-9276-11-15

**Published:** 2012-03-20

**Authors:** Khandsuren Lhamsuren, Tserendolgor Choijiljav, Enkhtuya Budbazar, Surenchimeg Vanchinkhuu, Diana Chang Blanc, John Grundy

**Affiliations:** 1 Chief Pediatrician, Bayanzurkh District Health Department, Ulaanbaatar City, Mongolia; 2 Deputy Director, Bayanzurkh District Health Department, Ulaanbaatar City, Mongolia; 3 Officer EPI team, National Centre for Communicable Diseases, Ministry of Health, Ulaanbaatar City, Mongolia; 4 Health and Nutrition specialist, UNICEF, Ulaanbaatar City, Mongolia; 5 Regional Immunization Specialist, UNICEF East Asia Pacific Regional Office, Bangkok, Thailand; 6 Public Health Consultant, Nossal Institute for Global Health, The University of Melbourne, Cambodia Office, Phnom Penh, Cambodia

**Keywords:** Health Inequities, Urban Health, Health System Strengthening, Mongolia, Reaching Every District

## Abstract

**Introduction:**

In recent years, the country of Mongolia (population 2.8 million) has experienced rapid social changes associated with economic growth, persisting socio-economic inequities and internal migration. In order to improve health access for the urban poor, the Ministry of Health developed a "Reaching Every District" strategy (RED strategy) to deliver an integrated package of key health and social services. The aim of this article is to present findings of an assessment of the implementation of the RED strategy, and, on the basis of this assessment, articulate lessons learned for equitable urban health planning.

**Methods:**

Principal methods for data collection and analysis included literature review, barrier analysis of health access and in-depth interviews and group discussions with health managers and providers.

**Findings:**

The main barriers to health access for the urban poor relate to interacting effects of poverty, unhealthy daily living environments, social vulnerability and isolation. Implementation of the RED strategy has resulted in increased health access for the urban poor, as demonstrated by health staff having reached new clients with immunization, family planning and ante-natal care services, and increased civil registrations which enable social service provision. Organizational effects have included improved partnerships for health and increased motivation of the health workforce. Important lessons learned from the early implementation of the RED strategy include the need to form strong partnerships among stakeholders at each level of the health system and in the community, as well as the need to develop a specific financing strategy to address the needs of the very poor. The diverse social context for health in an urban poor setting calls for a decentralized planning and partnership strategy, but with central level commitment towards policy guidance and financing of pro-poor urban health strategies.

**Conclusions:**

Lessons from Mongolia mirror other international studies which point to the need to measure and take action on the social determinants of health at the local area level in order to adequately reduce persistent inequities in health care access for the urban poor.

## Background

### The international context for health inequities

There are increasing reports of the public health significance of inequities in health access and outcomes. Inequity in health has been conceptualized as a "measure of difference" in access or outcomes based on social or economic exposures such as wealth, education level and location of residence. One analysis of equity and trends in coverage of maternal, newborn, and child health services in 54 countries between the years 1990 and 2006 found that, "in general, in-country patterns of inequality are persistent and change only gradually if at all" [1]. Many studies have indicated that these inequities are socially determined. That is, social exposures such as education, income level and rural residence are the best predictors of health service access and health outcomes for populations. Across Asia, evaluations from China, [2] India,[3] Indonesia, [4,5] Cambodia,[6] Vietnam [7] and Bangladesh [8] emphasize persisting health inequities in access to care and in poorer health outcomes for the socially disadvantaged. Additional studies highlight the multi-dimensional nature of poverty and ill health in urban slums including the heterogeneity of social structure, [9] and recommend a range of responses. These actions range from targeted medical interventions, to capacity building and skills transfer, to infrastructure development and improved networking among NGOs, municipal governments and private practitioners [10].

### The context for health and social inequity in Mongolia

A major characteristic of health inequities in the international context as outlined above is the impact of social and economic transition on health access. Mongolia serves as a prime example of this evolution. Since the early 1990s, following the end of the Soviet era, Mongolia (population 2.8 million) [11] has been undergoing a rapid social transition. Main elements of this transition include a rapid pace of urbanization, decentralization and a gradual opening up of the economic, social and political system. In line with this transition, there has been significant economic growth in recent years which has been averaging nine percent annually in the past five years (2003-2008). The gross domestic product (GDP) per capita has risen from US$456 in 2000 to an estimated $2,111 in 2010 [12]. However, the proportion of people living below the national poverty line has remained persistently high over the past 18 years [13], with the most recent estimate (2008) placing it at 35% [14]. Along with this pattern of uneven economic growth across social groups, there have been major demographic changes. The average annual growth rate of Ulaanbaatar's urban population during 2003- 2006 was approximately 3.6%, which was triple the rate of growth amongst the total Mongolian population (1.2%) and nearly four and a half times that of the aimag (provincial) centers (0.8%) [15].

During the peak of Mongolia's social transition, there were reports of increasing poverty leading to rapid migration to the cities [16]. Other factors linked to internal migration include loss of livestock and livelihoods of rural families due to natural disasters (severe winter snow storms referred to as the "dzud"), the search for new economic opportunity and lack of employment and social services in rural areas [17]. These socio-economic trends have resulted in the emergence of significant spatial inequalities in income and social opportunity [18]. Post socialist transition in the early 1990s was reported to have resulted in the "remorseless growth of inequality" in Mongolian society, with the wealthiest 20% of the population having eighteen times the income of the poorest 20% [19].

In addition to this modern evolution, there is a traditional pattern of internal migration within rural areas for the purposes of herding, as well as new patterns of migration associated with the emergence of an informal mining sector [20]. In the post socialist transition, reported declines in herding, farming and rural industries have been associated with declines in levels of social support provided to rural communities for services such as education, health, social welfare and state subsidies. These reductions have contributed to the high rates of urbanization as families move to cities to seek economic security, better social safety nets and employment opportunities.

In the local media, this growth in the urban population has been referred to as "the Great Migration" [21]. This migration to the city has placed great stresses on urban infrastructure. A recent assessment has indicated that in some districts of Ulaanbaatar, up to 90% of housing in some urban districts is in the traditional herding "ger" style of accommodation (traditional Mongolian housing on free land), with the gers clustered very close together without adequate water and sanitation systems [22].

Despite these challenges, in terms of health system development and health outcomes, there have been gradual improvements in maternal and child mortality rates in recent years. Between 1990 and 2008, the child mortality rate declined from 87.5 to 23.4 per 1000 live births, indicating that the Millennium Development goal (MDG) for child mortality reduction has already been achieved [23]. Family Group Practices, a mixed public-private model of primary medical care have been established and financed through a state capitation-based funding scheme and health insurance mechanisms [24]. The Family Group Practices are medical clinics staffed with five to eight doctors and nurses who provide a range of first-line preventive and curative care services, including immunization, reproductive health and communicable disease-control services. The Family Group Practice also acts as a referral point to higher-level facilities for more sophisticated curative care. A typical Family Group Practice covers a population of 17,000 in each sub-district or "khoroo" with a service radius of 5-8 km.

### The evidence for health inequity in Mongolia

Nevertheless, despite the evidence of some health gains, the consequence of these rapid social and economic changes has also demonstrated adverse health effects. Several studies have been published which highlight the acute health and social needs of the urban poor in Ulaanbaatar. A health planning study published in 2009 indicated that up to 67% of the capital's population now lives in a 'ger district' (i.e. districts characterized by 'ger' or traditional Mongolian housing on free land).

Many of these residents were defined in the study as being recent migrants, with up to 20% not being registered for social welfare, and who were therefore excluded from access to social health insurance benefits. These populations are living mostly in newly established ger areas -- in proximity of Ulaanbaatar's three biggest markets, in areas where student dormitories or child care centers are situated, and in locations with extremely under-developed infrastructure, particularly for water and sanitation. The main health risks for these populations were reported to be communicable diseases and lack of access to affordable health-care services [25]. Other published studies relating to health inequities in Mongolia includes data published by UNICEF in 2009, which indicates that the average poor family food consumption rate of USD $31 per month is one-third that of non-poor families (USD $93.50) [26]. Furthermore, a multi-indicator cluster survey published in 2005 indicated that the proportion of low birth weight was much higher among mothers with no or little education [27]. Estimates of maternal mortality rates in health facilities in Ulaanbaatar in a study conducted in 2007 indicated that rates had increased, and that these increased rates "might be associated with a higher birth rate and higher in-migration" [28].

Reports have also been published which indicate the limited capacity of the health system in Mongolia to respond to these recent social changes. Documented main challenges include the viability, staffing and quality of Family Group Practices, inadequate equipment and supply of essential pharmaceuticals at district hospitals, and major imbalances in investment between primary care (6% of national health care funding) and the hospital sector (70% of national health care funding) [29,30]. Although the Ministry of Health issued a decree in 2002 to provide medical services to all families including migrants and those unregistered, an in-depth study of access by the urban poor to social services indicated that family health clinics, which are financed on a per-capita basis, were not funded to cover these additional populations [31].

### Framework for taking action on health inequities - the reaching every district strategy (RED)

Inequities in access to health services can be analyzed from the perspective of the social determinants of health [32]. The social determinants of health perspective asserts that the fundamental causes of ill health are the basic conditions of daily living, necessitating a reorientation of health policy towards multi-sector action and public health policy.

However, although there is a significant body of literature on the analytic basis of social determinants of health, there is less on the role of community health services in taking action on, or measuring the impact of, the social determinants of health on health access. Broader analyses of initiatives to address health inequities in urban slums have highlighted the fact that there are specific differences in vulnerabilities among urban slum populations. As such, responsive action requires in-depth mapping and assessment of health needs, so that context specific solutions can be designed and implemented [33,34].

One such planning strategy that applies this context specific approach is the "Reaching Every District" strategy. The concept of the "Reaching Every District" approach (RED) was developed in 2002 by the World Health Organization (WHO), the United Nations Children's Fund (UNICEF) and global partners in order to increase immunization coverage for harder to reach populations [35]. Implementation in settings in Africa [36,37] and Asia [38] has demonstrated the effectiveness of the strategy in improving immunization coverage.

Originally intended as a problem-solving approach to boosting low immunization coverage, the global framework was adapted by the Government of Mongolia in 2008 to extend beyond provision of immunization services to include a more comprehensive package of health and social services. Specifically, the RED strategy in Mongolia was broadened to include maternal and child health services and civil registration, in recognition of the fact that that the barriers to high coverage were similar and cut across other interventions. Furthermore, it was recognized by local authorities that civil registration is required to sustain improved access to health and additional social services [39].

Of the five operational components of RED, Mongolia emphasized the following in the design of their "Reaching Every District Strategy Implementation and Guidelines":

• Conducting of mapping and barrier analysis for hard-to-reach populations

• Implementing delivery of a package of health and social services for the hard-to-reach

• Developing programs of supportive supervision and community partnerships to oversee implementation of the strategy

A national level workshop was conducted in 2008 to finalise the national guidelines. Bayanzurkh district in Ulaanbaatar was subsequently identified as the initial pilot area, due to its high rates of internal migration and urban poverty.

### Target populations for the RED strategy in Mongolia

Target groups for the RED strategy included the poorest, disabled, unregistered, remote and temporary populations, orphanage children, single mothers, child labor and school drop-outs. Family Group Practice staff in this study indicated that this group constituted approximately five percent of their catchment population. This is not to say that populations beyond this five percent do not have unmet health needs. Yet according to Family Group Practice staff in this assessment, these five percent are among the most vulnerable living under the strain of day-to-day emergencies. The civil registration office in Bayanzurkh District was reported in this assessment that 500 new registrations were being recorded per day, indicating continuing trends in rapid urbanization.

Previous estimates have indicated that unregistered populations can constitute up to 20% of the city or district populations [40]. There are four important implications for lack of civil registration status.

• Unregistered families do not have access to social welfare benefits; and

• Unregistered families are excluded from social health insurance, which translates into high out-of-pocket costs and avoidance or delays in treatment-seeking; and

• Unregistered populations are typically not reflected in the official local authority or Family Group Practice population registries, resulting in chronic underestimation of health service coverage; and

• Family Group Practices are not financed to provide health services to unregistered populations, thereby limiting staff incentive and capacity to actively reach out to these vulnerable groups.

In public health terms therefore, civil registration is highly significant in determining health access and ultimately, influencing health outcomes. A UNICEF report indicates that unattended births in Ulaanbaatar may be due to unregistered families being unable to access services because women who do not have adequate documentation can be denied medical services [41]. In one 2007 study of 680 migrants in two districts of Ulaanbaatar and two aimags (provinces), it was found that the majority of the migrants came from rural areas to the capital city in search of employment, to further their studies, or for improved access to social services. The study found that 40.6% of the migrants had not registered, with main reasons being given as not registered yet (30.8%), no transfer certificate from their administrative unit in the original area (20.1%), and lack of money (26.4%). The study also confirmed low access to medical services for this group due to poor awareness of their social rights and lack of registration [42].

## Aims and objectives of the RED assessment in Mongolia

The overall aim of the 2010 assessment was to determine the progress made in the implementation of the RED strategy in Bayanzurkh district. One specific objective was to document the additional children and clients reached for immunization and other maternal and child health services, and describe lessons learned from health planners and service providers with a view towards broader scale up of the RED strategy in Mongolia.

Based on these objectives, there are four assessment questions that were explored and are captured in this paper:

1. What have been the main barriers (community, programmatic and health system) to health access for the very poor?

2. What have been the main activities implemented under the RED strategy in 2009?

3. What have been the main effects of the RED strategy in improving access to immunization and maternal and child health (MCH) services for hard-to-reach populations?

4. What are the main lessons learned for health policy and planning, particularly in relation to the issue of inequities of health access and outcomes for the urban poor?

## Methods

### Data collection and analysis

#### Site selection

Ulaanbaatar City consists of 17 districts, including Bayanzurkh. Bayanzurkh district is comprised of 24 sub districts or "khoroos" and has a population of 228,379. Bayanzurkh is considered a 'ger district' because more than 90% of the population lives in a ger (traditional mobile dwelling) [43]. The RED strategy was piloted over a 12 month period in No. 13 Khoroo in Bayanzurkh District. This selection was based on the following criteria: (a) Newly classified area with migrant populations (b) area with remote or dense populations (b) Area where high-risk populations are typically concentrated such as large markets, charity centers, student dormitories or places with poor infrastructure (particularly water and sanitation).

#### Data collection and analysis

A qualitative approach was applied for the assessment that included literature and program documentation review, and key informant interviews (n = 25), Family Group Practice group discussion (n = 6) and district health team meetings (n = 2). A questionnaire was developed to guide interviews with key informants (see annexure), exploring the assessment questions outlined earlier. Respondents included health providers (doctors and nurses) in Family Group Practices, district level health managers and social development officers, staff at the district civil registration office, city health staff and national health planners and policy makers.

In addition to the above research conducted in Bayanzurkh district, a tool was developed to analyse community, financial and other system barriers to health access. This tool incorporated mapping of unreached populations and was applied in other areas of Ulaanbaatar City where the RED strategy had not yet been implemented.

For the barrier analysis, a one-day training workshop was conducted to prepare five teams of district and Family Group Practice assessors to:

• Create a map of the hard-to-reach or unreached populations in one Family Group Practice catchment area; and

• Define the community impediments to health services access (for immunization and maternal and child health) as well as assess the health system and programmatic impediments to health services provision; and

• Develop a strategy for improving access and service delivery for the most vulnerable population, including identifying actual or potential local area health partnerships.

The Family Group Practice assessors presented their findings to the wider plenary of assessors (i.e. Family Group Practice health staff from other locations who participated in the assessment). The findings were analyzed using the barrier analysis framework as previously described and structured according to main research questions as outlined in section 1.6. In some locations, the teams visited the sites of the unreached populations and discussed in detail with resident Family Group Practice health staff the main impediments in health care access for the very poor. The case studies, presented in the Additional file 1 attached to this paper, were selected and summarized from these findings.

This case study approach was critical in illuminating the contextual reality of the local area, thereby better informing local area planning responses. Knowledge gained from these case studies contributed to a more nuanced understanding of the ways that the social determinants of health can influence health service access and thereby outcomes.

As this was an internal evaluation requested by the Ministry of Health, and respondents included health workers only and not community members, ethics clearance was not required nor requested.

### Limitations of the assessment

There are three overall limitations of this assessment.

Firstly, the overall purpose of the assessment is formative - that is, the assessment was intended to guide overall directions of the program and demonstrate potential for scale-up. The focus in the assessment is therefore mainly on aspects of process.

Secondly, barriers to health access for the urban poor are examined from the perspective of health managers and providers only. Community and client perspectives on health access barriers (aside from literature references) were not the subject of this assessment.

Thirdly, due to the particular characteristics of the target population, it can be difficult to define a clear denominator on which to base population coverage. Additionally, the RED strategy is not implemented on a district-wide basis, but includes mapped areas and sub-communities with mobile populations. Lack of clarity of denominators limits the capacity of the assessment to measure progress from a baseline. Nevertheless, as will be seen in the results section, data has been collected on additional populations reached, and the population denominator in the RED Strategy implementation area.

Due to the fact these were principally local area qualitative studies with the above mentioned limitations, findings cannot be generalized with high levels of confidence to other areas of the country. However, there was sufficient evidence generated from the assessment to inform strategic and operational directions in the medium-term for a gradual scale-up of the RED strategy in Mongolia (see section 3.2.5).

## Results

### Barriers to health access in Ulaanbaatar

The case studies in the Additional file 1 to this paper outlines the social context for health inequities in Ulaanbaatar, as described by district health staff. The summaries of these case studies demonstrate the complexity and diversity of residents' circumstances, and provide a rationale for a local planning strategy that responds to community health and social needs. Seasonal migration to summer camps, urban migration to city fringes, and daily mobility through central market areas are just some of the mobility patterns described in these case studies.

Just as the pattern of mobility is varied, so is the pattern of vulnerability. Poverty, disability, unregistered status, the unemployed, dormitory students, the homeless and school drop-outs are some of the categories of the vulnerable described in these case studies, with a wide disparity in living circumstances including apartment blocks, ger neighborhoods (traditional Mongolian housing), riverside dwellers and street dwellers. It has been reported elsewhere that some of the urban poor live in summer houses and then retreat underground for the winter [44].

One of the main barriers to health access described in the following case studies was the lack of health system preparedness to meet the needs of the vulnerable, particularly with respect to the lack of financing of health care services for unregistered populations and the insufficient numbers of health staff to serve them.

The second case study in the Additional file 1 of this paper examines in more detail the convergence of community, system and financial barriers to health access for the disadvantaged in urban Ulaanbaatar.

For primary level care, prevention services were reported to be free for children, pregnant women and the elderly. In February 2002, the Ministry of Health issued a decree that all Family Group Practice staff must provide services to their clients in the catchment areas regardless of registration status. However, "the budget issue was left untouched" [45]. This assessment identified that for treatment, a checkup fee is required and drugs must be purchased. If unregistered, clients have to pay at the hospital level and provide the full cost of medicines. This being the case, the poor then are observed by providers to delay seeking preventive care. This delay in health-care seeking behavior means that the poor then present more frequently as delayed emergency cases or severe illness. One Family Group Practice staff member remarked:

"There was one family that had twins one year, and 11 months later they had another set of twins. The father is a severe alcoholic. He has no ID card, apart from an old socialist passport. Due the absence of registration, this family now has a 4 million debt to the hospital. The problem is, how can health problems be solved when social problems are not?"

Another stated that for the vulnerable population, "there is no motivation to go to prevention services if you have no money for treatment. So some referrals do not go, and may not be admitted as they have no money."

One Family Group Practice reported that up to 60% of the population does not have the capacity to pay for essential medicines, and a minority of the population has no money at all. So although the capitation-based funding, health insurance and civil registration mechanisms have established the basis for social protection for the majority of the population, the system clearly lacks sufficient depth and breadth of entitlements to draw in the most vulnerable groups of the population. These groups in some locations are now effectively "outside the system." This finding is consistent with that of a previous study, which determined that health services charge clients for services when they do not have medical insurance and that medicines were too expensive for the poor [46].

Consistent in the discussions across case studies is the widespread perception by Family Group Practice health staff that they are not adequately financed and directed to actively search for and include the vulnerable populations into the health care system (particularly unregistered populations). This perception of the rigidity of the system is reinforced by the observations amongst Family Group Practice health staff and district managers that the growth in health resources (human resources and financial resources) is not keeping pace with the acceleration in the numbers of urban poor. In one Family Group Practice visited, the population had doubled in the last 10 years but health personnel remained the same, suggesting that health policy and planning is lagging well behind the social transition in Ulaanbaatar.

### Effects of RED strategy implementation in Bayanzurkh district and lessons learned

#### The RED process in Bayanzurkh

Implementation commenced in 2009 through funding support from UNICEF. The strategy was implemented in No. 13 Khoroo in Bayanzurkh at a total cost of 17 million Tkg (USD $ 14,166) for one year and with continued financing by UNICEF for 2010. The objective of the strategy, as defined by Bayanzurkh planners themselves, was to "detect target populations, determine their encountered problems, and implement a package of health and social services" [47]. Specific services to be offered included immunization, ante-natal care, integrated management of childhood illness, nutrition, civil registration and social support such as emergency food provision [48].

In 2008, a mapping exercise was conducted with district health planners and Family Group Practice health staff. Planners identified the location of the most at-risk populations, as well as documented the health system and community factors that caused limited access for vulnerable groups. The health planners and Family Group Practice health staff subsequently identified priority activities and costs to reach those populations [49].

Following launch and issuing of a District Health Administrative order, a training program was conducted for heads of family clinics, Bayanzurkh district and sub district governors, specialists and the administrative team of Bayanzurkh District Health Center. Five supervisors were nominated from the district level.

Initially, the training participants decided to take a "sample selection" at community level of the households in high-risk areas, as they had identified through the mapping exercise. But subsequently, they discovered that it was necessary to walk house-to-house in order to detect the vulnerable groups. One health team reported that after this switch in tactic, the health team started to find unimmunized children. The increased numbers of families detected who required health and social services was described by the respondents as "unexpected."

There is one nurse available for each sub-khoroo (or "section") Under the RED strategy, the nurse walked each month through the sub-khoroo identifying new clients (normally dedicating three days a month). She did not provide services on these walks, but instead requested new clients to attend the health facility or come to the registration office. The person was registered at the health facility or at the Governor's office, and the information was shared between the Family Group Practice and the civic authorities. In addition to meeting community members in their homes, nurses liaised with NGOs, section leaders and social welfare and registration staff at the office of the khoroo Governor to discuss the situation of the socially vulnerable. Nurses transmitted their monthly data to the district level where the analysis was conducted on a quarterly basis. Data sheets recorded the names of staff members from the khoroo and numbers of new clients contacted, classified into target groups (e.g., the disabled, temporary residents or unregistered populations, the elderly or orphans without carers, single mothers, the unemployed, school drop-outs, unimmunized children and unmonitored women for reproductive health care). The district compiled data on services provided and conducted on-site supervisory visits. Specific activities conducted by supervisors included monitoring data quality, problem-solving of implementation issues and meeting with the target groups.

#### Effects on health access

In one khoroo where the RED strategy had been implemented in Bayanzurkh District (Khoroo No 13), the nurses identified an overall population of 22,726 in the target catchment. Using agreed criteria of vulnerability, the nurses then identified a sub-population of 8,708 highly vulnerable, of whom 3126 were children under the age of 15 years and 708 were elderly. Vulnerable groups included the poorest, disabled, unregistered, remote and temporary populations, orphanage children, single mothers, child labor and children having dropped out of school. Other categories included elderly and disabled persons without caregivers and the homeless. Malnutrition was also identified to be an issue in this sub-population, with 144 children being referred to a nursery for care and rehabilitation. Under the initiative, the following *additional*contacts were made (that is, numbers over and above existing contacts):

a. Additional immunized children 219

b. Additional registered populations at Family Group Practices clinics 2485

c. Additional ANC care contacts 508

d. Additional family planning contacts 1047

e. Additional emergency food supply provision 515

Table 1 outlines these additional contacts with reference to the wider target population.

**Table 1:** Findings of Improved Access, Mongolia RED Assessment 2010

Intervention	At Risk Target Population	Numbers at Risk Reached	% of Identified at Risk Reached by the RED Strategy	General Target Population in RED Catchment Areas	% of Additional Population Reached through the RED Strategy
Additional immunized children	489	477	97.55	3126 (children 1-15)	15% of the target children for immunization

Additional registered populations at Family Group Practices clinics	2598	2485	95.65	8708(Total Population in RED Area)	28.5% of total registered population at the family clinics

Additional ANC care contacts	515	508	98.64	5616(Total ANC Contacts in RED Area)	9% of all ANC Contacts

Additional family planning contacts	1047	1047	100.00	96560(Total Family Planning Contacts in RED Area)	1.08% of all family planning contacts

Additional emergency food provision	Not yet fully assessed	515	Not yet fully assessed	8708(Total Population in RED Area)	5.9% of Target population

There is a total target population in the RED trial area of 8708, and an immunization target of 3126 (children aged 1-15). A subset of this group was targeted by the RED Strategy planners. An additional 477 children have been reached, representing 15% of the total target group for immunization in the catchment area of the RED Strategy. There were 2485 additional community members who were registered at the Family Group Practice, which represents 28.5% of the total target population. As this is a high migration area, this is consistent with other sources cited in this paper, which indicate there are vicinities in the city with 20% of its residents who are unregistered, and hence who are less likely to access health insurance and social welfare benefits.

#### Effects on staff motivation

In some locations where the RED strategy was implemented, Family Group Practice staff reported increased motivation, linked to their closer involvement with the community. The nurses in one location expressed that, although many of the problems of the vulnerable population were overwhelming and could not be solved by the nurses; the nurses felt a high level of satisfaction and achievement in helping the families to solve some of the problems. Others commented that before the RED strategy they could only see one perspective, but that afterwards they could appreciate social vulnerability from a different point of view.

A district health planner commented that their workload was previously focused on facility care, but that the RED strategy had opened up the possibility to work outside the facility in the community.

"Before I worked in the facility only - Now I work in the community and so now my work is more interesting and challenging."

But limits to motivation were also noted. Nurses considered their incentives for community involvement to be too small, given the extra burden of work invested in monthly walks through the community, at times strenuous. Others suggested that the strategy may not be sustainable as the communities expand further under the pressures of internal migration. In this situation, some Family Group Practice staff maintained that it may not be possible to walk house-to-house detecting vulnerable populations. One manager commented that nurses were doing social work which was considered "other people's work". In order to meet the social needs of the population, respondents indicated that incentive systems should be devised for local authorities (section leaders) and social workers in order to increase civil registration at the Family Group Practice. Despite these limitations, overall, early implementation of the RED strategy has been reported to raise motivation of Family Group Practice practitioners and of district managers.

### Effects on health partnerships

The significance of the RED strategy in stimulating new partnerships (or strengthening existing partnerships) was reinforced at various levels of the health system.

At *Central Level*, lessons learned from the RED strategy have been absorbed in the design of the health system strengthening approach, for which Mongolia has been awarded funding from the Global Alliance for Vaccines and Immunization during the timeframe 2010-2012 [50]. Funding for the RED strategy from various sources will be managed by a working group at the National Center for Communicable Diseases (NCCD), with inputs from a higher-level policy working group at the Ministry of Health. In April 2011, Ministerial Order #154 was issued recommending national scale up of the RED strategy for health sector strengthening.

At the *City level*, health planners and managers are monitoring the strategy, and are supportive of proposals to scale-up the strategy and widen participation at the district level in the RED working group.

At the *District level*, a RED working group has been established comprising the District Health Director, Social Development staff, an accountant and other district health staff. In recognition of the value and need for health and social sector partnerships, it is now proposed that the RED working group be widened to include civil registration representatives and others from social welfare agencies.

At the *Community level*, the involvement of an NGO employee as a liaison officer with the Family Group Practice practitioners highlights how partnerships are evolving. This NGO assists vulnerable populations with acute social needs such as emergency housing. A partnership was also strengthened with a nursery, where 144 children had been referred for nutritional rehabilitation, after having been detected through the RED strategy. In terms of the office of the Khoroo Governor (where civil registration and social workers are based) and section leaders, it was reported that this is an area where partnerships could be strengthened. Clearly, the fact that that civil registrations had resulted in extra client contacts for health services amongst the vulnerable groups all point towards improved communication within the community as a result of the RED Strategy. As one of the Family Group Practice nurses commented:

"The RED strategy is there to solve problems - this means running after the social needs of the population so their health needs can be met".

#### Effects on health policy

Following assessment of the RED strategy and trial implementation in two urban districts and three provinces (aimags) between 2008 and 2010, Ministerial Order #154 was released in April 2011 by the Ministry of Health outlining the management arrangements for national scale-up of the strategy [51]. These management arrangements include the establishment of inter-sectoral working groups from national to soum/khorro levels (sub-district levels) of the administrative system in order to improve health and social access for the poor. In 2012, the level of scale-up of the strategy will extend to four urban districts and five rural aimags (provinces).

The contents of Ministerial Order #154 reflect important trends in equity-focused health planning and policy-making, and include the following main components:

• Definition of a monitoring and evaluation framework that describes baseline measures for planning and management processes for pro-equity planning, and health and social service outcomes (particularly increased uptake of civil registration and social welfare benefits);

• Definition of a national management structure for the RED strategy, with establishment of an inter-sectoral committee at national and city level, and the establishment of RED working groups at sub-district level that are coordinated by local government;

• Definition of core functions of RED working groups incorporating barrier analysis, identification of target groups, oversight of implementation, conducting baseline surveys and progress reporting.

## Discussion and conclusions

### The potential of the RED strategy to address inequity and social disadvantage

The application of the RED strategy in Mongolia is an example of active social management (through the health sector) in order to correct social disadvantage. The observations by health managers and providers in this assessment capture the challenge of responding adequately to the health needs of the population without also addressing their social problems. Interviewees in this assessment underlined the notion that the transition to the market economy and related patterns of internal migration had loosened social bonds, weakened traditional community leadership and strained extended family ties, thereby negatively impacting on health for many vulnerable groups.

This isolation presents itself in a number of ways. These include being "outside the health system" in terms of lack of financial capacity or knowledge to access health services, being "outside the administrative system", as measured by lack of civil registration, being "outside the economy" as measured by unemployment or low-income status, and, most importantly, being "outside of any social network" as measured by lack of any "carer" status (single mothers, orphans or elderly without care-takers, school drop-outs). Ill health in the Mongolian urban setting is therefore more than a consequence of being low-income, but reflects a "compound effect" [52] of poverty, unhealthy daily living environments, social vulnerability and isolation.

This RED strategy assessment has underlined the extent to which individuals and families in the lowest socio-economic groups lack the social and behavioral pathways of social influence, engagement and social support that characterize "networked" healthy societies [53]. It is conceivable that the RED strategy, as a method of "instrumental" social support for access to health and some social services, is able to assist with the tangible needs of the population by linking them better to mainstream social agencies (including NGOs, local authorities and government services).

### National context - service delivery models, partnerships and health care access

The establishment of the Family Group Practice system in Mongolia, along with development of health insurance mechanisms and a capitation-based model of funding have provided a solid foundation for the development of the national health system [54]. However, based on the outcomes of the RED strategy in Bayanzurkh district, it is quite clear that this solid foundation, though a necessary condition, is currently insufficient to meet the needs of the most vulnerable groups in society. These groups have in some cases fallen "outside the system," with high levels of social isolation amongst them. Furthermore, based on the case studies of health access barriers, there is convincing evidence that significant minorities of the population are not accessing health and social services at all.

The service delivery model of the Family Group Practice in many ways is built around the concept of primary medical care, and less perhaps on the concept of primary health care. Although mandated by Ministerial Order #154 to service the health needs of the registered and non-registered elderly, children and pregnant women, some Family Group Practices are simply not funded or organized to reach such groups, providing little motivation to actively search for them. Even with clear Government orders, it is all too easy for the poor and powerless to fall outside the scope of mainstream service provision. The awareness raised among health workers in Bayanzurkh, that after house-to-house search, an "unexpected number" of vulnerable clients were identified seems to confirm this tendency. This realization has important implications for the financing and organization of health care service delivery for the very poor.

The Family Group Practice is of course a "Family" Group Practice and not a "Facility" Group Practice, so additional community participation mechanisms will ensure that families remain the focus of the health care system. In practice, this means stimulating more community-focus in daily operations which partly target wider social issues that affect access to health services (school attendance, social isolation, unemployment, disability care, substance abuse, nutrition). For this reorientation to take place in practice, local area mechanisms for participation by all stakeholders will need to be modeled in order to formalize the networks and develop procedures for resolution of community health problems. The nurses of Bayanzurkh demonstrated that this reorientation must come from a range of organizational and social actors, and not only from the health sector itself.

On the other hand, as participants in this assessment indicated, there are limits to the Family Group Practice's capacity and function to adequately respond to the social determinants of health. Nevertheless, the Family Group Practice still has the potential to develop and strengthen social networks for health. In order to re-engage vulnerable families with the health and social system and have long-term impact, the structure supporting the Family Group Practices has to change and broaden to include other responsible civic actors including district health authorities, Khoroo Governors Offices and section leaders, NGOs and volunteer networks and the families themselves. Some ideas from this RED strategy assessment of how these partnership mechanisms could work are detailed in the additional file attached to this paper.

The complex array of social determinants that contribute to social isolation and related poor health, are clear arguments for integrated approaches to service delivery. The cases of social exclusion outlined in this paper would respond best to participatory or "partnership" methods of health governance that are more inclusive and expressive of the health rights of disadvantaged social groups.

### Looking to solutions - improving health and social services access through the RED strategy

The experience of the RED strategy in Mongolia raises one of the perennial questions of primary health care practice. Should health services promote demand for fixed facilities or should health workers "outreach" to these communities? The difficulty with unique emphasis on a fixed-facility approach is that health issues become contextualized in terms of clinical health responses only. There is less opportunity for community networking to stimulate improved health, and a tendency of health care providers to "personalize" what are in fact social health issues. The fact that many of the health managers and providers in this assessment easily identified the underlying social causes of poor health access for their catchments gives testimony to the fact that a more networked health strategy will be required to make a sustainable positive impact on health outcomes.

The lack of "connectedness" between health administrations and local authority was recently noted in an international review of health inequity studies. Over a 20 year time frame, it has been demonstrated that only 17% of the studies and interventions described roles for municipal government in the reduction of local health inequities, and that studies and interventions demonstrate a "pervasiveness of 'behavioral' and 'biomedical' perspectives [55]. One other review has highlighted the fact that local governments often lack the financial and expert capacity to address urban problems, resulting in the lack of representation of the needs of the poor in local urban governance and planning [56].

The constraints of Mongolia's health system and local government organization raises real questions about the efficacy of such a heavy emphasis on "contractual" models of health care delivery, certainly in a society marked by high levels of income inequality and mobility. The "chaos" described by one of the respondents to characterise the level of community disorder, combined with the comments by a central planner of things seemingly "being out of control" highlights the need for both stronger central resource commitment and decentralized "steering" of the sector to ensure that health service delivery is more inclusive of the needs of the very poor in local areas. The fact that large numbers are falling "outside the system" indicates the need for putting in place practical procedures for mapping, tracking and registering populations and for strengthening systems of community partnerships to effectively reach these populations and respond to their needs. Table 2 illustrates the contrasting approaches of contractual models of management with models of governance informed by a social accountability approach.

**Table 2:** Enhanced Contractual Models of Health Service Governance through Lens of Social Accountability, Mongolia RED Assessment 2010

Contractual Models of Care Based on Essential Services Package	Application of Social Accountability Lens
Capitation-based funding	Capitation Based Funding (funding per capita) in addition to "Special Funds" Arrangements for Marginalized Populations

Outcomes-based funding	Both Process and Outcomes Based Funding (for example financing of community participation and partnership building in addition to activities supporting achievement of coverage targets)

Planning and financing for officially registered populations	Financing for officially registered populations as well as financing of the unregistered based on population estimates provided through micro-planning data

Processing health entitlements through facility-based operations	Promoting population entitlements to health and social service access through active participation and networking in vulnerable sections of the community

Provision of primary medical care	Promotion of primary health care through community partnering and networking with municipal authorities, NGOs and community leaders (in addition to delivery of standard package of medical benefits)

Facility-based operations	Facility-based operations combined with active community search and engagement of the vulnerable for equity of access to health and social services

This strengthening of partnerships in a decentralized context as outlined above and in the section above is reflected in Ministerial Order #154 on national scale-up of the RED strategy. This order is highly illustrative in terms of new approaches to health system strengthening being adopted in Mongolia.

*Firstly*, the transition of management of the RED strategy from the National Immunization Program to the Department of Planning is in recognition of the fact that, in order to address persistent inequities that have a basis in the social determinants of health, a health system strengthening approach that embraces a wider package of health and social services will be required.

*Secondly*, RED in Mongolia is now driven by stronger partnerships between system planners (local government, health planning and finance) and program planners (immunization and maternal and child health). The value of the partnerships between system and program planners is that the former have the capacity to mobilize resources and encompass a wider health and social services agenda, and the latter (particularly from immunization as demonstrated by the success of the RED strategy internationally) have the capacity to build upon successful operational strategies at community level.

*Finally*, the new policy on RED in Mongolia also illustrates the developing role of local authorities and social welfare agencies in partnering with the health sector to address inequities in access to health and social services. In fact it is local authorities, with social welfare and health sector participation, who will oversee the strategy at the local level [57].

The RED scale-up, facilitated under the national directives of the Ministry of Health and supported through UNICEF and the Global Alliance for Vaccines and Immunization, is bolstered by a wider set of evolving partnerships across local government, NGO and social welfare agencies. This indicates much better prospects for sustaining the strategy in the longer term.

However, despite the health access improvements and structural gains achieved under the RED strategy, some important limitations of the strategy need to be noted, particularly with respect to its capacity to quantify the increase in health service access coverage for the urban poor. As has been observed in earlier sections of this article, the particular characteristics of urban poor populations include their mobility (on a seasonal or daily basis) and lack of registration status. Notwithstanding these methodological difficulties, this assessment establishes the limitations of qualitative methods, as well as the constraints of effectively capturing the quantitative outcomes of the RED intervention. This indicates the need for a more "mixed-methods" approach in future assessments, with consideration being given to conducting cross-sectional household surveys in targeted areas to balance the case study findings.

## Conclusion

The findings captured in this RED assessment on the limitations of the health care delivery system in Mongolia are entirely consistent with the conclusions in international literature as alluded to in the introduction of this paper. Specifically, without a more consciously pro-poor approach to targeting health services, health systems will not gain ground in reducing in-country disparities in health care access [58]. The findings also illustrate the extent to which the health sector has struggled to replace heavily centralized administrative systems with more inclusive and participatory models of governance, particularly as these systems apply to the health and social needs of the very poor. Reviews on the role of social determinants of health policy in reducing health inequity conclude (as does the RED strategy) that integrated or multilevel approaches are required not only at policy-making and political levels, but also most critically at local area level [59,60].

One of the important features of Mongolia's RED strategy is that it is a pro-poor social management plan. This mainstreaming of the poor into the health and social system is not just symbolic - the mainstreaming is in fact instrumental, with civil registration being a cornerstone to bringing tangible benefits. This includes linking marginalized populations to social welfare and NGO agencies for shelter, management of malnutrition, and improved access to immunization and family planning services. This encapsulation of health and social concerns into a social management plan provides an "opportunity rope" for populations to achieve their optimal potential for development and encourages fuller participation in the community fabric.

The diverse social context for health in an urban poor setting also necessitates a decentralized planning and partnership strategy, but with central level commitment towards policy guidance and financing of pro-poor urban health strategies. This paper provides some early evidence that the implementation of the RED strategy has resulted in improvements in health access, health partnerships and staff motivation in Bayanzurkh district. This being the case, ongoing health system and social research will be required to deepen qualitative understanding of how to overcome barriers in access to social services by the very poor in urban settings. Finally, given the challenge in tracking populations and defining population denominators in the urban poor context, more attention will be required to design operational strategies and research methods which quantify baselines and measure outcomes for improving health care access for the urban poor.

## Competing interests

The authors declare that they have no competing interests.

## Authors' contributions

KL and TC led design and implementation of the strategy at the local level. EB provided national oversight and technical guidance for implementation. SV provided technical guidance at country level and facilitated design, participated in field research and technically reviewed drafts. DCB provided technical inputs into design and substantially revised manuscript drafts. JG conducted a literature review, participated in field research and drafted the initial paper. All authors read and approved the final manuscript.

## Annexure: Question guideline RED assessment Mongolia

(for interview with key informants from health services and Local Authorities)

Date: Location:

1. What are the main barriers to access for hard-to-reach populations in this area?

a. System barriers (Human Resources, finance, transport and communications, management)

b. Community barriers (knowledge, attitudes, practices, socioeconomic factors)

c. Programmatic barriers (drug and vaccine supply, cold chain, IEC materials etc.)

2. What activities are currently implemented for reaching hard-to-reach populations?

3. What have been the main effects of program implementation up until now?

4. What have been the main lessons learned to date, and what are the continuing gaps in service access?

5. Based on these observations, what needs to be done (recommendations) to improve access?

## Supplementary Material

Additional file 1 **Case studies Mongolia red assessment 2010**. Click here for file
