# Supplementary material for: Taking action on the social determinants of health: improving health access for the urban poor in Mongolia
Source: Int J Equity Health. 2012 Mar 20;11:15. doi: 10.1186/1475-9276-11-15 (PMC3349495; doi:10.1186/1475-9276-11-15)
Supplement: Additional file 1 — Case studies Mongolia red assessment 2010. [file 1475-9276-11-15-S1.DOCX]

**ADDITIONAL FILE: CASE STUDIES MONGOLIA RED ASSESSMENT 2010**

**Case Studies in the Social Context for Urban Health, Mongolia RED Assessment 2010**

*CASE 1* This *is a summer camp* with a rural location and high rates of fluctuation in population – these communities are based in the northern hills of Ulaanbaatar and are characterized by ger accommodation and large distances between gers. Migration here is seasonal with a large population influx from the city in the summer period, when the inhabitants increase from the officially registered number of 12,000 to up to 30,000. There are also migrants in this location who reside here during the winter months. All the population reside in gers and 65% are considered vulnerable by the Family Group Practice (FGP). The FGP is located outside the Khoroo territory. There are also remote areas that are hard-to-reach with no mobile immunization teams. The remotest area is 45 kilometres from the FGP among the mountains and valleys in the North.

*CASE 2* This *is a more central urban area* with a series of large day and night markets and very high rates of daily migration. In this location, there are poor residents who live in apartments and others living temporarily in hostels. This area is also the location of child laborers, temporary shopkeepers, and small vendors. High rates of homelessness and alcoholism are also observable in this area. Many migrants and temporary residents live and sell goods near the open markets.

*CASE 3* This *‘Ger Khoroo’* (sub-district with concentrated ger neighborhoods) has very high rates of internal migration, as there is ample room for the gers to expand westwards. Here many of the residents are migrants and live towards the northern part. The population has doubled in the last 10 years as the Khoroo expands. Approximately 10% of the population is identified as “the poorest” –unemployed, single mothers, families with many children, the disabled and those with chronic diseases.

*CASE 4* In another “*Ger Khoroo”*, the community consists of 11% residing in apartment blocks and the remaining 89% living in gers. 1,900 people out of a total of 12,000 do not live at a registered address and there are high rates of migration into the community. There is a lack of clear boundaries between this Khoroo and the neighboring one. Where the administrative affiliations are unclear is where the vulnerable people are concentrated. In this area there are many hostels. This increases density of temporary residents in poor living environments that are insecure. Students and young couples are crowded into these locations. There are no partnerships projects with NGOs and no volunteers. There are no immunization units so therefore residents have to travel seven to eight kilometers to another Khoroo for services. There is no active seeking of un-immunized and unregistered populations by the health staff.

*CASE 5* is inhabited by *river-side dwellers* who are very poor and reside in temporary shelters made from boxes, scrap and iron along the banks of the river. This is a Khoroo located south of Ulaanbaatar near the airport. The vulnerable here include the homeless, unemployed and unimmunized children. The population grew from 8552 in 2007 to 9323 in 2009, with the vulnerable estimated at 20% according to data from the local authority office. These vulnerable were said to include isolated children (98), unregistered residents (37), temporary residents (76) and school drop-outs (50).

**Case Study of Social Disadvantage in Ulaanbaatar, Mongolia RED Assessment 2010**

*The Population* of this Khoroo is 9760 and is divided into six sections. This area includes five open markets including a night market. In addition to the resident and temporary resident population, up to 10,000 people visit from other parts of Ulaanbaatar City every day to frequent the markets. Ninety percent of the population lives in gers, and ten percent in apartment blocks. The level of mobility in the population is confirmed by registration data. Registration records at the Khoroo Governor’s Office (9,760) and Family Group Practice (11,071) are markedly different, which seems to confirm the high level of mobility and influx of temporary residents. 47% of the population is classified as poor by the local authorities (that is, with a monthly disposable income of less than 74000 Tkg or the equivalent of 59 USD$). Family Group Parctices estimate poverty in a different way. They estimate poverty rates at 60%, as this is the proportion of the population that cannot afford to buy medicines.

*The Environment*: There are many migrants and temporary residents living and selling goods near the open markets, with over 10,000 visitors coming daily. Waste management is very bad in this area and open waste proliferates. There are three deep wells for the population and water transportation services are available. The migrants put the gers anywhere they want, pay no electricity and move again when electricity inspectors cut access. The living conditions are very poor with large numbers of homeless and street alcoholics who are exposed and sometimes die in the winter cold. Other vulnerable population groups include the unemployed, people who rent hostels, the disabled and those who live in manholes. There are also child beggars, with children being employed to provide labor for the vendors. There are daily residents who come to collect the daily rubbish and sell it. They are considered as outsiders and are not eligible to receive health services, but the staff of the Family Group Practice (FGP) tries to register and provide services to them.

*The Health System*: The ratio of population to doctor is very high due to this large influx of temporary visitors. The standard ratio is 1800 residents for every 1 doctor, but now it is 4000 to each doctor with high staff turnover. There are unimmunized children because some of the population does not even know the location of the FGP.

*What needs to be done*: District and FGP planners identified needs to establish a permanent Immunization Unit at the FGP so that community members do not have to travel as far as the District to seek services. The need was identified to address the social and environmental determinants of health, in particular environmental risk and action, including improvements to water and sanitation. The need was identified to develop team building at the grassroots level including training and deployment of volunteers. A cross-sector Task Force or Working Group for RED was recommended to be established. This would involve an increase in cooperation between the FGP and the Governors Office, District Governor’s office and the Social Development Department.

**Lessons Learned for Health Partnership Mechanisms, Mongolia RED Assessment 2010**

At the district level in Bayanzurkh, a RED Working Group has been established in order to coordinate and provide oversight to the implementation of the RED strategy. Members include the District Health Director and Health Team, Social Development staff and Civil Registration staff. This multi-sector participation in the RED Working Group is the recognition by leaders of the impact of social determinants on health services access and health outcomes. Such mechanisms are in the early stage of implementation, and there is potential for replication of this model at the FGP/community level.

At this level, as the experience in Byanzurkh has demonstrated, there are many actors and stakeholders for health. These include the FGP practitioners, NGOs, Office of Khoroo Governor, section leaders, volunteers and the families themselves. It would not be difficult to imagine a replication of the RED Working Group at community level, with establishment of a community participation mechanism in order to:

- Provide a forum for resolution of important public health concerns of the community
- Promote of civil registration
- Coordinate health and social services for the new arrivals and very poor
- Discuss methods and systems for motivating section leaders and volunteers to communicate health and social problems to the relevant agency
- Coordinate NGO activities

Ideally, such a community participation model should be chaired by the Khoroo or Soum (sub-district) Governor. Administrative orders, terms of reference and additional support to develop capacity would be required in order to establish and trial such a mechanism, which would need follow-up evaluation.
